# Routine Immunization Consultant Program in Nigeria: A Qualitative Review of a Country-Driven Management Approach for Health Systems Strengthening

**DOI:** 10.9745/GHSP-D-15-00209

**Published:** 2016-03-25

**Authors:** Meghan O’Connell, Chizoba Wonodi

**Affiliations:** ^a^Johns Hopkins Bloomberg School of Public Health, International Vaccine Access Center, Baltimore, MD, USA

## Abstract

**Background:**

Since 2002, the Nigerian government has deployed consultants to states to provide technical assistance for routine immunization (RI). RI consultants are expected to play a role in supportive supervision of health facility staff, capacity building, advocacy, and monitoring and evaluation.

**Methods:**

We conducted a retrospective review of the RI consultant program’s strengths and weaknesses in 7 states and at the national level from June to September 2014 using semi-structured interviews and online surveys. Participants included RI consultants, RI program leaders, and implementers purposively drawn from national, state, and local government levels. Thematic analysis was used to analyze qualitative data from the interviews, which were triangulated with results from the quantitative surveys.

**Findings:**

At the time of data collection, 23 of 36 states and the federal capital territory had an RI consultant. Of the 7 states visited during the study, only 3 states had present and visibly working consultants. We conducted 84 interviews with 101 participants across the 7 states and conducted data analysis on 70 interviews (with 82 individuals) that had complete data. Among the full sample of interview respondents (N = 101), most (66%) were men with an average age of 49 years (±5.6), and the majority were technical officers (63%) but a range of other roles were also represented, including consultants (22%), directors (13%), and health workers (2%). Fifteen consultants and 44 program leaders completed the online surveys. Interview data from the 3 states with active RI consultants indicated that the consultants’ main contribution was supportive supervision at the local level, particularly for collecting and using RI data for decision making. They also acted as effective advocates for RI funding. In states without an RI consultant, gaps were highlighted in data management capacity and in monitoring of RI funds. Program design strengths: the broad terms of reference and autonomy of the consultants allowed work to be tailored to the local context; consultants were often integrated into state RI teams but could also work independently when necessary; and recruitment of experienced consultants with strong professional networks, familiarity with the local context, and ability to speak the local language facilitated advocacy efforts. Key programmatic challenges were related to inadequate and inconsistent inputs (salaries, transportation means, and dedicated office space) and gaps in communication between consultants and national leadership and in management of consultants, including lack of performance feedback, lack of formal orientation at inception, and no clear job performance targets.

**Conclusions:**

While weaknesses in managerial and material inputs affect current performance of RI consultants in Nigeria, the design of the RI consultant program employs a unique problem-focused, locally led model of development assistance that could prove valuable in strengthening the capacity of the government to implement such technical assistance on its own. Despite the lack of uniform deployment and implementation of RI consultants across the country, some consultants appear to have contributed to improved RI services through supportive supervision, capacity building, and advocacy.

## Abstract (precis)

Despite challenges in material and managerial support, some state-level consultants appear to have improved routine immunization programming through supportive supervision and capacity building of health facility staff as well as advocacy for timely dispersion of funds. This country-led, problem-focused model of development assistance deserves further consideration.

## INTRODUCTION

Nigeria has had a complex history of immunization dating from the 1970s/1980s. Bilateral and multilateral aid agencies were active supporters of immunization efforts during that time, but aid funding was compromised during a period of political turbulence, which led donors to cut funding in the country. Under civilian rule from 1999 onwards, the National Programme on Immunization (NPI) was established with a focus on polio. NPI was subsumed into the National Primary Health Care Development Agency (NPHCDA) in 2007 and international donors reentered the arena, but for many years routine immunization (RI) coverage performance undulated.^1^

During the last decade, there have been gradual improvements in national coverage for vaccines such as bacille Calmette-Guérin (BCG) for tuberculosis, the third dose of diphtheria-tetanus-pertussis (DTP3), polio, and hepatitis B, according to 2014 estimates from the World Health Organization (WHO) and the United Nations Children’s Fund (UNICEF).^2^ Strengths in Nigeria’s current RI system are most apparent at higher government levels. Strong support for RI is evident from NPHCDA and the Federal Ministry of Health (FMoH), and funds for vaccine procurement have been consistently included in the federal budget. In addition, after several years of challenges, Nigeria has succeeded with interrupting polio transmission, and in October 2015 the country was certified polio free after 1 year of no polio cases. Overall, great achievements have been made in reducing mortality rates among children under 5, from 201 per 1,000 live births in 2003 to 157 in 2008, and to 128 in 2013.^3^

Despite these major improvements, vaccine-preventable diseases still constitute a significant proportion of deaths in under-5 children,^4^ and recent progress occurs in the context of ongoing systemic challenges. The 2013 Demographic and Health Survey (DHS) showed staggeringly low national vaccination coverage rates, with only 38.2% of 1-year-olds vaccinated with DTP3 and 42.1% for measles.^3^ Furthermore, only 25.3% of 1-year-olds received all basic vaccinations and 20.7% received no vaccinations at all.^3^ A 2012 landscape analysis of the Nigerian RI system identified a number of central weaknesses that included inadequate transportation, improper cold chain management, financing barriers, stock-outs, poor accountability and performance management, poor integration of RI services with broader primary health care services, and unreliable administrative data.^4^ Although program strengths and weaknesses vary across states due to the decentralized structure of governance in Nigeria, these weaknesses represent overarching challenges that must be tackled at the systems level.

Despite major improvements in reducing under-5 mortality in Nigeria, vaccine-preventable diseases are still an important cause of deaths.

As a lower middle-income country, Nigeria is quickly outgrowing its eligibility for international development assistance. Considered a frontier market economy by the World Bank,^5^ in 2014 Nigeria was ranked as the largest African economy after a rebasing calculation almost doubled the gross domestic product (GDP). While as a whole this is a positive development, critical donor support for health from organizations such as Gavi, the Vaccine Alliance, is determined in part by a country’s gross national income (GNI) per capita. By doubling the GDP overnight, Nigeria became eligible to move into a transition phase for Gavi funding starting in 2017, which entails a gradual phasing-out of all funds over a 5-year period. As the international community moves beyond the Millennium Development Goals to the Sustainable Development Goals and the Nigerian government becomes progressively more responsible for the cost of health programming, an emphasis must be placed on problem-driven, adaptive health programs that are designed and managed by Nigerian stakeholders and firmly rooted in country realities.^6^

This article describes and reviews an RI consultant program in Nigeria that is in many ways such a problem-driven, iterative, and adaptive approach to aid programming.^7^ The program is aligned with ongoing efforts in the country to improve accountability in RI by clarifying the roles of governmental players and increasing transparency of reporting, supervision, and evaluation mechanisms across different levels of government. The specific objective of this study was to understand the implementation, strengths, and weaknesses of the RI consultant program in Nigeria to inform future strategies. The study was part of a portfolio of projects run by the Johns Hopkins International Vaccine Access Center (IVAC) in Nigeria under the Gavi-funded Vaccine Implementation Technical Advisory Consortium (VITAC). The projects aim to provide evidence and technical assistance (TA) to support the Nigerian government to introduce new vaccines and strengthen RI systems.

## PROGRAM DESCRIPTION

Since 2002, the RI consultant program has been jointly funded by Gavi and NPHCDA, a parastatal of the Nigerian FMoH. Through this program, NPHCDA is meant to deploy 1 consultant to each of the 36 states in the country as well as to the federal capital territory to serve as technical support for RI. Although there are variations, the size of the job that consultants are tasked with is quite large. Nigerian states have an average of 4.9 million people and 21 local government areas (LGAs).^8^ As part of the decentralized government structure, each state has its own constitutionally designated funding and budget and operates its own health services with staff that are state employees. The overall goal of the RI consultant program is to support states to implement RI effectively and to strengthen RI systems by improving delivery, data quality, and capacity of workers at the health facility and LGA.

The Nigerian RI consultant program is meant to deploy 1 consultant to each of the 36 states and to the federal capital territory to provide technical support for RI.

The design of the RI consultants program is unique in that it is not pure development assistance in the traditional sense of being fully funded by a donor government, multilateral agency, or private agency. NPHCDA and Gavi funds have had an equal share in the cost of recruiting and maintaining the RI consultants, but the proposal and design process was country driven and activities were designed by Nigerian stakeholders in response to an expressed need to better coordinate Gavi support at the state level.

Several components of the design distinguish the program’s TA model:

1. Program is designed and managed by domestic actors and tailored to the local context.
2. Consultants are locally experienced staff recruited for long-term contracts.
3. Consultants are selected by domestic actors.
4. Work is primarily field based.
5. An emphasis is placed on advocacy and networking skills.^9^
6. TA is focused on resolving broader problems within the public sector (e.g., capacity building for data quality assurance).
7. Work is oriented toward outcomes that are uncertain and difficult to measure (e.g., judicious use of funds, capacity building, and advocacy).

The scope of the program has been gradually scaled-up in an iterative fashion, from 6 consultants in 2002 (1 in each geopolitical zone) to 17 consultants who coordinated between 1 and 3 states in 2008. By 2012, the program had intended on scaling-up to 37 consultants (1 per state plus 1 to the federal capital territory), but execution of the proposed 1-consultant-per-state model has not been entirely successful. At the time of data collection (September 2014), there were only 23 consultants deployed to the states.

Recruitment of RI consultants occurs at the national level. Contracts are awarded on an annual basis and consultants are hired as state officers for a specific posting. Although their contracts are reviewed annually and subject to termination, consultants are meant to be long-term contractors of NPHCDA and are meant to reside in the state in which they are deployed. Candidates must be 35–55 years of age with a familiarity with Nigerian public-sector health systems; many consultants previously held high-level positions such as Commissioners for Health in their states and are well respected within their field.

Consultants are strategically placed as external players to the governmental health system to position them to be advocates for RI. Although they exist outside the government health system, the way in which consultants work through state and LGA structures varies by state. In some states, they work closely with the existing state RI team but are also able to work as more independent entities when necessary. Consultants are expected to spend the majority of their time in the field and to visit a minimum of 3 LGAs per month to conduct supportive supervision. Within their states, RI consultants are meant to interact primarily with the existing state RI team and LGA-level staff.

### Intended Roles

**FIGURE 1.**
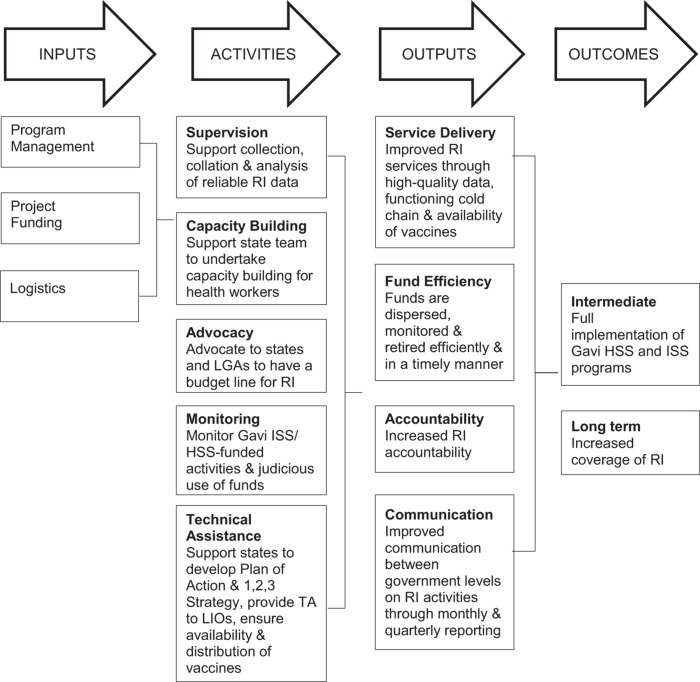
Logic Model of the Nigerian RI Consultant Program Abbreviations: HSS, health systems strengthening; ISS, immunization support system; LIO, local immunization officer; LGA, local government area; RI, routine immunization; TA, technical assistance.

According to their terms of reference (TOR), the intended role of RI consultants is to act as a liaison between national, state, and local governments to build the capacity of state-level actors to implement RI effectively. See Figure 1 for the program logic model. The consultants are meant to strengthen RI systems through:

- **Supportive supervision** of health facility staff in RI data management and quality assurance
- **Capacity building** for health workers at the LGA level
- **Advocacy** to the state for the appropriate use of Gavi funds, and to states and LGAs for a budget line for RI
- **Monitoring** the implementation of all Gavi immunization support system (ISS) and health systems strengthening (HSS) activities and the judicious use of Gavi funds within the state
- **Technical assistance** in implementation of RI activities within the state

The goal of the consultant program is to strengthen RI systems through supportive supervision, capacity building, advocacy, and monitoring of funds.

## METHODS

### Study Design

We conducted a retrospective review from June to September 2014 using a mixed-methods study design consisting of semi-structured qualitative interviews and online surveys. A study advisory group comprised of international, national, and state-level experts was formed to provide guidance on the data collection and analysis approach and to determine analysis outputs most useful for NPHCDA.

### State Selection

**FIGURE 2.**
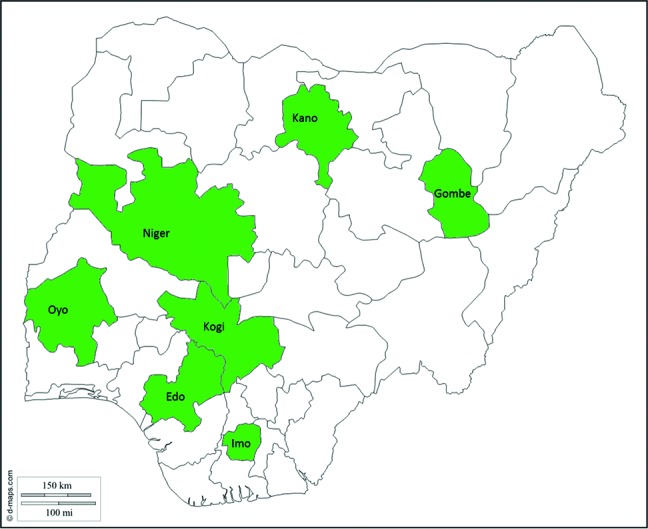
Nigerian States Selected for Qualitative Data Collection

We selected 7 states (Edo, Imo, Kano, Kogi, Niger, and Oyo) from all 6 geopolitical zones to represent diverse programmatic settings while balancing security concerns (Figure 2). State selection criteria were based on RI performance (high/medium/low DTP3 coverage), urban/rural, current IVAC or consultant’s partnerships, accessibility, and security (Table 1). Although the unweighted average of the DTP3 coverage for the selected states was 58%, which was 20 percentage points higher than the national average of 38%, 2 of the selected states, Kano and Gombe, had lower than average DTP3 coverage at 19% and 36%, respectively. States with and without RI consultants were included to determine what, if any, gaps were being filled by the presence of the RI consultant.

**TABLE 1:** State Selection and Criteria

State	2013 DTP3 Coverage^a^	Urban/Rural	IVAC Relationship	Active Consultant	Airport Accessible	Security Concern
Kogi	High (75.9)	Rural	None	Yes	No	Low
Niger	Medium (67.3)	Semi-Urban	Strong	Yes	No	Low
Gombe	Low (36.0)	Rural	None	No	Yes	Medium
Kano	Low (18.9)	Urban	Strong	Yes	Yes	Medium
Imo	High (83.1)	Semi-Urban	Strong	Yes	Yes	Low
Edo	High (79.6)	Semi-Urban	None	Yes	Yes	Low
Oyo	Low (47.7)	Urban	None	No	Yes	Low

Abbreviations: DTP3, third dose of diphtheria-tetanus-pertussis; IVAC, Johns Hopkins International Vaccine Access Center.

a DTP3 coverage: Low: <50%, Medium: 50%–74%, High: ≥75%. Source of coverage data: 2013 Nigeria Demographic and Health Survey.

### Study Procedures and Instruments

We developed the qualitative interview guides based on intended roles identified through the consultants’ TOR (Box 1) and on input from key stakeholders including RI consultants and the national NPHCDA Gavi Focal Person. The discussion guides were refined iteratively throughout the data collection process to ensure gaps were filled and questions were appropriate.

**BOX 1. Terms of Reference and Key Deliverables for Gavi Routine Immunization Consultants in Nigeria**

**Terms of Reference**

1. Advocate to states and local government areas (LGAs) to have budget line (Basket Fund) for routine immunization activities.
2. Support states and LGAs to develop Plan of Action for Routine Immunization at the state and the LGA level. The officer should, on a monthly basis, analyze the status of implementation of this plan of action, provide feedback to the state team, and report to the national level (NPHCDA).
3. Support and ensure implementation of the 1,2,3 Strategy in their state of assignment.
4. Support state team to undertake capacity building of health workers in their state of assignment.
5. Support the collection, collation, and analysis of reliable routine immunization data on a regular basis through supportive supervisory visit to health facilities and LGAs and participation in the monthly data quality checks and data quality self-assessment.
6. Provide technical support to the monthly meeting of local immunization officers (LIOs) in their state of assignment.
7. Support the state team to ensure that routine immunization vaccines are available in the state and that they are properly distributed to LGAs in a timely manner.
8. Monitor the status of implementation of all the activities in the Gavi immunization support system (ISS) and health systems strengthening (HSS) objectives and report to the national level on a quarterly basis.
9. Monitor the judicious use of Gavi funds at all levels in their state of assignment.
10. Any other activities as directed by the Director of Disease Control and Immunization at NPHCDA.

**Key Deliverables**

1. Monthly report on activities conducted including routine immunization performance in state of assignment, status of implementation of 1,2,3 Strategy, minutes of meeting of LIOs, funds available/released for routine immunization activities in the state, vaccine status by LGAs, and status of implementation of state plan of action.
2. Training report.
3. Report on status of utilization of Gavi funds in state of assignment.
4. Quarterly report on status of implementation of Gavi ISS/HSS activities.

Independent research consultants trained in survey administration administered the interview guide to each participant. In some cases, 2 or more participants were interviewed together using the same qualitative interview guide. Each interview took 30–60 minutes and included probing on the following domains:

- Roles and responsibilities of RI consultants
- The extent the program has been implemented as planned
- Main clients
- Monitoring and supervision
- Strengths and weaknesses
- Acceptability

Interviews were conducted at 3 tiers of government (national, state, LGA) as well as at the facility level, with tailored interview guides for each type of respondent. All participants provided oral informed consent, and information collected from the interviewees was de-identified to maintain confidentiality.

We also conducted 2 online quantitative surveys—one of state program leaders (e.g., state immunization officers, directors of primary health care) and the other of RI consultants—to complement findings from the qualitative interviews. Questions for both surveys covered topics on the perception of consultants’ roles, visibility, and impact as well as on interactions between all state RI players, the presence of budget lines and reporting structures, and effectiveness of advocacy activities. We used Survey Monkey to send and collate survey responses from target respondents across all states in Nigeria and from the national level.

### Study Participants

Participants were eligible to be included as an interviewee if they were RI consultants, had been involved in the RI consultant program design or were knowledgeable of its implementation, or had regularly interacted with RI consultants. We used purposive selection of respondents to ensure representation from different job positions and levels of government. Individuals had a minimum of 1 year of experience in their current role, unless they occupied a unique position and no other alternative existed, in which case they were not excluded if they had less experience. In general, we interviewed RI program leaders, managers, and implementers from the government and from partner organizations.

For the online survey of consultants, we sent the instrument to the 23 current consultants and to 13 former consultants for whom we had current contact information. The second survey was sent to 48 program leaders.

### Analysis

All interviews were audio recorded after obtaining verbal consent from respondents. Interview tapes were transcribed verbatim, and each transcript was hand coded independently by at least 2 members of the study team to identify salient themes. Coded transcripts were entered into Atlas.ti qualitative data analysis software, which was used to query the qualitative data to examine meaningful content and interpret the data in terms of identified themes. Codes were derived inductively from the proposed logic model (Figure 1) and grounded in the themes emerging from the data. We developed the final codebook through team discussion and consensus. For the online survey, we assessed prevalence of key opinions and perspectives on the role of RI consultants and triangulated that data with in-depth information from the interviews.

### Ethical Approval

This study was approved by the Johns Hopkins Bloomberg School of Public Health Institutional Review Board and the National Health Research Ethics Committee of Nigeria.

## FINDINGS

### Background Characteristics

In total, we conducted 84 qualitative interviews with 101 participants. Of the 84 interviews, 70 had complete and high-quality recorded data. Thus, we conducted qualitative data analysis on 70 in-depth interviews with a total of 82 individuals from 7 states and from national-level stakeholders based in the federal capital territory.

Among the full sample of interview participants (N = 101), the majority (66%) were men, with an average age of 49 years (±5.6) (Table 2). Most participants had a graduate degree, and 38% had a postgraduate degree. Most participants (63%) were technical officers, but several other roles were also represented (22% consultants, 13% directors, 2% health workers). All participants were highly experienced in RI, with an average of 23 years (±8) of work experience in health care and 17 years (±8.5) of specialized experience in immunization.

**TABLE 2:** Demographic Characteristics of Interview Participants

	All Interview Participants (N = 101)
Gender, No. (%)	
Male	67 (66)
Female	34 (34)
Age, mean (SD), years	49 (5.6)
Employment level, No. (%)	
National	7 (7)
State	53 (52)
LGA	41 (41)
Respondent Type, No. (%)	
Director	13 (13)
Active RI consultant	5 (5)
Other consultant (e.g., WHO consultant)	17 (17)
Technical officer	64 (63)
Health worker	2 (2)
Years in position, mean (SD)	6 (4.9)
Years in immunization, mean (SD)	17 (9.1)
Years in health care, mean (SD)	23 (8.6)
Obtained postgraduate education, No. (%)	38 (38)

Abbreviations: LGA, local government area; RI, routine immunization; SD, standard deviation; WHO, World Health Organization.

The overall response rate for the qualitative survey was 63% (N = 59), with 42% (n = 15) for the consultants survey; of the consultants, 80% (n = 12) were current consultants. The leadership survey had a response rate of 76% (n = 44). Program leaders had worked, on average, 6 (±4) years in their current job, and 25% of the respondents were state immunization officers, 19% classified themselves as national facilitators (n = 9), and 14% (n = 6) were national immunization officers. The low response rate for consultants was likely due to technical challenges with Internet connectivity in rural states as well as low motivation and incentive to respond among former consultants.

The level of implementation of the RI consultant program differed by state. Of 7 states visited for this study, 5 had RI consultants posted while 2 did not. Of the 5 states with a posted RI consultant, 3 states had consultants that were present and visibly working in the state while 2 states did not currently have consultants or the consultants were not generally on the ground and were not well integrated within the RI state team or RI activities.

Of the 7 states visited for the study, only 3 states had RI consultants who were present and visibly working.

### Strengths and Weaknesses of the RI Consultant Program

We iteratively defined the consultants’ success based on qualitative evidence that they were engaged in RI work within their state of assignment and executing their TOR to the extent possible given their individual circumstances. In this sense, a successful consultant was one who, according to interviewees, was consistently on the ground and engaged in their expected role (advocacy, supportive supervision, capacity building, monitoring Gavi funds, TA) as it related to their states’ needs.

The 3 states that had RI consultants present and visibly working were distinct in that they actively engaged consultants, allowing them to be highly integrated within the state RI team through facilitated multi-stakeholder engagement.^6^ In these states, implementation of activities generally aligned well with the TOR, and consultants were identified as highly respected and highly motivated individuals, capable of coordinating effectively with the state RI sector and with NGO partners.

In states with active RI consultants, these consultants were integrated well within the state RI team.

Strengths and challenges identified by the interviewees and survey respondents are summarized below according to the key roles of the consultants defined in their TOR, followed by strengths and challenges in the overall design of the program. See Box 2 for a summary of the key findings.

**BOX 2. Main Findings From Qualitative Interviews**

- In states that had active and present routine immunization (RI) consultants, implementation of activities was generally well aligned with the consultants’ terms of reference.
- Consultants’ main role was as supportive supervisors at the local government area (LGA) level; they also acted as effective advocates for RI funding and played a key role in quality assurance of RI data.
- Program design strengths included recruiting consultants who were highly committed and motivated, familiar with the local context and language, and well respected within their field.
- Key challenges in program implementation were related to inadequate and inconsistent inputs (salaries, transportation means, dedicated office space) and gaps in management of the consultants (monitoring and supervision).
- In states without an RI consultant, capacity for data management and Gavi fund oversight were identified as the primary gaps.

### Supportive Supervision and Capacity Building

Qualitative interview data from the 3 states with an active RI consultant program showed that consultants contributed to improved delivery of RI services through supportive supervision that improved the availability of high-quality data and improved functioning of the cold chain. The main activity of the RI consultants was universally expressed in these 3 states to be supportive supervision to the LGAs. This supportive supervision was intentionally focused on correction of inefficient or ineffective processes at the LGA and facility level in order to strengthen the operational system of facilities and improve delivery of RI services. Respondents at each level indicated that RI consultants played an integral role particularly with undertaking outreach services, cold-chain maintenance, and general problem solving. This supportive supervision went hand in hand with capacity building through on-the-job training (either planned or ad hoc during field visits), which was focused on the transfer of skills to LGA-level staff to build competency in providing RI services.

RI consultants reportedly provided supportive supervision for collecting and using RI data for decision making.

The most important supportive supervisory role played by consultants was identified as supervision for collecting, collating, and analyzing reliable RI data within the health facility. Interviewees indicated that, when engaged in their state, RI consultants facilitated quality assurance of RI data reported from health facilities and LGAs to the state through trainings and data quality checks. This process intentionally focused on building LGA capacity to collect and use data for decision making. Respondents indicated that consultants fed coverage data up to state leadership and that they also used these data to map unmet need, identify unsatisfactory performance, and address challenges to reaching target populations, thereby enhancing the performance of facility outreach services. Improved quality of data and increased competency and education of LGA staff were reported as the main impact from this supportive supervision.

In states without an RI consultant, the most frequently identified gap associated with the lack of supportive supervision was assistance with data management. The success in data quality improvement achieved in states with a consultant was succinctly illustrated by an LGA Cold Chain Officer:

*The health facilities are working, but the problem we are having with them is the data. They are not producing data correctly, and that data speaks for the LGA. If they don’t do it right, we go there and see that the coverage is low. With [the RI consultant’s] presence in the state, they are going out to correct them. It improves their data … it is one of their greatest achievements.*

Unfortunately, due to limited resources available to consultants, the ability to conduct supervisory visits was often constrained by lack of transportation and logistical support. Consultants often had to either use their personal vehicles or “hitch a ride” with other state RI team vehicles conducting fieldwork. This likely restricted the quantity and quality of the supportive supervision that could have occurred had consultants been provided with reliable transportation means.

Limited transportation means constrained the ability of consultants to conduct supervisory visits.

### Advocacy

Interview respondents from states with an active RI consultant program indicated that advocacy activities conducted by consultants were useful and were associated with fund efficiency (timely dispersion and monitoring of funds) and increased problem solving at the state and local level.The skills profile and gravitas of the RI consultants, who have many years of experience and strong professional networks, make them uniquely placed to conduct advocacy within their state for the appropriate use of Gavi funds and to state and LGA leadership for a budget line for RI. When active in their state, both qualitative and quantitative results indicate that consultants appear particularly well suited to facilitate the resolution of RI funding challenges by communicating directly to leadership within the state, such as the Commissioner, Executive Secretary, and Director of Primary Health Care, whereas other workers within the state team are not in a position to request these meetings. Importantly, the topic of advocacy was not constrained by a predetermined policy agenda but was focused on general problem solving within the consultants’ state. A State Immunization Officer indicated that the RI consultant in his state had “the skill to convince the chairman or be able to stand before the chairman,” while a Local Immunization Officer explained:

This year he has also done 3 advocacies to the LGA chairman to resolve problems. The last time was when there was power failure at the LGA cold store due to non-payment of bills, he advocated to the Chairman of the LGA and the problem was resolved immediately.

The experience and gravitas of the RI consultants made them uniquely placed to conduct advocacy activities.

Additionally, having familiarity with the local context and speaking a local language were seen as assets for advocacy. One Director of Primary Health Care explained:

*One of the strengths is that the [RI] consultants are from the state so they also understand the internal dynamics of the state and some challenges. They are also able to help because there are some people they can also reach to give support to the program due to their status and also they are team players; that one is a big advantage.*

### Monitoring of Funds

One of the biggest challenges since the inception of Gavi funding has been timely retirement of funds in the states, specifically of ISS funds. Nigeria received ISS funds, which primarily supported routine immunization, for 4 years (in 2001, 2002, 2006, and 2007).^8^ Study participants indicated that the retirement of 2007 ISS funds was still outstanding at the time of this research, which has acted as a bottleneck for disbursement of new funds.

While there were striking differences between states with and without consultants in terms of information reported on Gavi fund tracking, qualitative results indicated that some consultants could facilitate the implementation and correct use of Gavi funds, but not their retirement. On the other hand, 48% (n = 21) of quantitative survey respondents said that consultants facilitated both release *and* retirement of Gavi funds (Table 3). The discordance between these results is likely due to a lack of understanding by leadership of the consultants’ actual activities. Retirement of Gavi funds remains a problem and Gavi consultants seem ideally placed to facilitate this process. However, retiring funds has not been part of the consultants’ TOR, and they were not granted the required signatory power to retire Gavi funds, making it unlikely that they have played any significant role in this process.

**TABLE 3:** Findings From Quantitative Survey of Nigerian RI Consultant Program Leaders (N = 44)

	No. (%)
How useful are your interactions with RI consultants?	
Very or somewhat useful	34 (77)
Neutral	10 (23)
Not very useful or not useful at all	0 (0)
Is the RI consultant visible and actively working on RI activities in your state?	
Yes	32 (73)
No	10 (23)
I don’t know	2 (5)
To what extent is the RI consultant involved in decision making for RI at the state level?	
Very involved	17 (39)
Somewhat involved	11 (25)
Not very involved or not involved at all	12 (27)
I don’t know	4 (9)
The work of the RI consultant has a large impact on improving RI services in my state	
Strongly agree or agree	31 (70)
Neutral	8 (18)
Strongly disagree or disagree	5 (11)
Does the RI consultant have influence to facilitate the release and/or retirement of Gavi HSS and ISS funds?	
Yes, release only	1 (2)
Yes, retirement only	2 (6)
Yes, both release and retirement	21 (48)
No	11 (25)
I don’t know	9 (20)
How much influence does the RI consultant have on the Gavi HSS and ISS funds?	
A lot or some	20 (45)
Not much or none at all	13 (30)
I don’t know	11 (25)
Are there clear performance targets for the RI consultant in your state?	
Yes	14 (32)
No	4 (9)
I don’t know	26 (59)
Do you or anybody in the state evaluate performance of the RI consultant?	
Yes	9 (20)
No	30 (68)
I don’t know	5 (11)

On ensuring the correct use of Gavi funds, qualitative results indicated that a communication gap existed between consultants and their national-level leadership such that they could perform this task but at times did not have enough information to do so. There was no consistent mechanism in place from the national level to inform RI consultants when Gavi money was dispersed to their state, so they were not always aware of the movement of funds. Despite it being a main component of the consultants' TOR, only 18% (n = 8) of survey respondents said that consultants had “a lot of influence” on use of Gavi HSS and ISS funds.

Notwithstanding the communication gap, in states where consultants were active and present, it was very clear that the consultants made an effort to track the use of Gavi funds, ensure guidelines were followed, and report misuse to state leadership for correction. Although modalities for monitoring use of funds were not structured and uniformly applied, a few consultants were able to track fund use at the LGA and health facility level and advocate for health facilities that did not receive funds. In contrast, interviews with participants in states without an RI consultant showed that this type of Gavi fund oversight was not apparent, or the interviewees specifically identified it as an area that needed attention. When asked if RI consultants made sure that Gavi funds were used for the specific programs for which they were provided, a State Deputy Director in a state without a current RI consultant (*but that had one previously*) explained:

*They [the consultants] release the funds to local government immunization officers—these funds are for the intensification of RI, for logistics support for the smooth running of RI programs at the local government level—and they have done that well. When they were here, we felt the impact—our RI coverage increased. But when they left, it decreased. So they have done well, to my own knowledge.*

### Overall Program Design

#### Strengths of the Design

There are several components of the program design that might have facilitated engagement of consultants in their key roles (i.e., advocacy, supportive supervision, capacity building, and monitoring of Gavi funds) within their states. First, the broad TOR and autonomy within the state allowed work to be tailored to the context, and it was clear that consultants could adapt to their states’ needs. Second, the national Gavi Focal Person was a staff member of NPHCDA and facilitated the placement and integration of consultants in their respective states. This allowed consultants to work as part of the state RI teams that composed the state and LGA RI government structures. Although part of the state team, consultants could also draw on their external status to work more independently within the state when necessary. Furthermore, recruitment that prioritized advocacy and networking skills enabled a powerful player to exist within RI to act as a direct advocate for problem solving at the local level and to feed RI information up to state decision makers. In addition, we saw that selection of indigenes for long-term, field-based contracts allowed relationship building that guided progress.

#### Challenges to Implementation

While the consultant’s independence from the state team was strategic and had benefits for advocacy, many implementation challenges were due to this external position. There was consensus among all interview participants in all states that the dominant challenge to implementation of the RI consultants program was funding—specifically, receipt of timely remuneration for consultants. Nearly all respondents regardless of role reiterated that there was a lack of timely payment; in some cases, payment had been delayed for more than a year. Lack of designated office space and, more importantly, a means of transportation, were also cited as major challenges across the board to achieving success in RI consultants’ work. Although a portion of the consultants’ stipend was meant to cover transportation costs, it was universally expressed that their remuneration was not sufficient and that transport without a vehicle was a key challenge. If most of their work was seen as supervisory, having no means of transportation posed a major hindrance to their effectiveness.

Timely remuneration of consultants was a major challenge of the program.

A second key challenge in implementation was related to gaps in management, specifically the monitoring and supervision of RI consultants from the national and state levels. Specific gaps in management included:

- No structured feedback on monthly consultant reports from the state or national level
- No field-based monitoring/supervision from the state or national level
- No co-management between state and national levels on consultants’ activities
- No formal orientation with the state team
- No clear job performance targets

Gaps in supervision of consultants also constrained the program.

Although annual performance evaluations with real repercussions for subpar work indicated that there was no longer tolerance for poor performance, the oversight infrastructure to incentivize good performance did not exist.

## DISCUSSION

Evidence for the effectiveness of the RI consultant program is limited. Many consultant posts (14 of the 37) were not filled, some consultants were inactive, and supervision of the program was weak. Despite these structural challenges, we found many positive elements in the midst of substantial difficulties. When staffed with qualified and motivated people, the RI consultants were useful in supportive supervision and advocacy for RI. A few consultants were also able to track ISS funds and report misuse to state leadership in line with their TOR.

Despite structural challenges, some RI consultants appear to have improved RI programming through supportive supervision and advocacy.

Outsourcing critical RI program functions (supervision, cold chain maintenance, data management, monitoring of financial flows, etc.) risks creating consultant-driven dependencies, a concern underscored by our findings that continuity of the consultants is an issue in many states. However, we found that consultants have the potential and ability to be more than just a temporary helping hand for RI in their states and that their external position was a strategic positioning rather than a temporary solution. Although internal government champions are critical to the RI advocacy process, external advocates are also necessary. Many of the RI consultants were former senior health officials. This experience allowed them to navigate the government system from outside and solve problems that a more junior or less well-connected person could not. Furthermore, their professional experience brought gravitas to their role as RI advocates. Unlike typical consultancies, the program is meant to be a long-term solution. Although contracts are reviewed annually, the consultants are not placed in states as temporary or short-term staff. The risks to continuity at the time of the study were due to structural challenges in funding, not to flaws in the program design.

The RI consultant program design represents a TA model that is partially supported by an external donor and managed by a domestic partner, targeting systems strengthening through supervision and capacity building for improved service delivery in RI. In practice, we saw enthusiasm for the model for improving RI, but an inability of some consultants to overcome structural challenges to effect change. Overall, there is a lack of guidelines or models of TA implementation^10^ and, until recently, a lack of case studies of programs that are locally led, problem-driven, iterative, and adaptive.^7^ The absence of guidelines may work in favor of avoiding decontextualized best practices that assume one model fits all, but it may also have contributed to a plethora of TA models on the ground that may or may not be effective, are not standardized, and have not been evaluated. The findings from this qualitative study contribute to the literature on TA by synthesizing the components of the program that were well received, either by design or by default, and that were aimed at strengthening an RI system within an environment that is challenging politically, economically, and environmentally.

### Recommendations

Based on our research, we put forth the following recommendations for improving the RI consultant program:

1. Improve the timeliness of payment of the consultants and consider increasing remuneration. Also consider providing the consultants with additional inputs, such as a vehicle for transportation and dedicated office space, to facilitate successful implementation of their key roles.
2. Enhance the existing monitoring and supervision system to include:
  - Joint planning with the state team and leadership on consultants’ priority activities, targets, and deliverables. Targets should be based on an assessment of the gaps and the solutions that the consultant is best positioned to address.
  - A more detailed analysis of consultants’ monthly reports with a structured mechanism for feedback
  - Field-based monitoring of consultants’ activities within the state from the national level and/or monitoring of consultants’ activities by the state leadership
  - Structured reporting by the consultants to a specified person in the state leadership
  - Appointment of an external (non-NPHCDA), national-level focal person to coordinate activities, communication, and monitoring of consultants, as well as to interface with the NPHCDA Gavi Focal Person
  - A quarterly or annual review meeting with consultants and other partners to document lessons learned and inform future strategies
3. Implement an in-person orientation with the state team at inception of the RI consultant into the state to introduce the stakeholders, align expectations within the state, and allow for joint work planning between the national and the state level.
4. Provide a structured mechanism to disseminate information on the disbursement of funding to consultants’ states to empower consultants to play a more active role in monitoring fund utilization and management.
5. Provide RI consultants with training on effective mentoring. Although the RI consultants had many mentoring relationships, training could help improve and systematize the RI consultant program across the different states.

To be more actionable, key stakeholders should discuss these recommendations, identify responsible parties for each component, and agree on the most effective method to operationalize each point.

### Limitations

There are some limitations to this study. First, the study was almost exclusively qualitative, which limited the scope and ability to address important questions. Because most of the data were obtained through interviews, the information collected may be susceptible to interview bias. A more in-depth desk review that examined available meeting minutes or documentation in states, LGAs, and health facilities would have added depth to our results. Additionally, a more in-depth analysis of the political drivers of variance in the RI consultant rollout, implementation, and attrition within different states would have added to our results. Lastly, it is intuitive that a study of a larger scale, one that examined programmatic effects in more than 7 states, would have added value and insight to our analysis.

The primary limitation in the interpretation of our results is our inability to definitively link the work of consultants to system-wide effects and coverage rates within the states and hence an inability to determine the impact of the program. This information would have provided more insight into the value of the program and an ability to produce stronger recommendations for potential future directions of the program.

## CONCLUSIONS

While weaknesses in managerial and material inputs affect current performance, the design of the RI consultant program in Nigeria is a unique model for development assistance that aims for transfer of resources and technical skills. Supportive supervision, capacity building, and advocacy were seen as the most valuable roles provided by the RI consultants to improve delivery of RI services; these roles seem to have facilitated collection and use of high-quality data, improved fund efficiency, and increased problem solving. If improvement in management processes occurs, consultants may be successful despite systemic challenges common in the difficult political context of Nigeria.

## Notes

**Peer Reviewed**

## References

[1] World Health Organization [Internet]. Geneva: World Health Organization; c2015 Reported estimates of BCG coverage; last updated 2015 Sep 8 [cited 2015 Nov 15]. Available from: http://apps.who.int/immunization_monitoring/globalsummary/timeseries/tscoveragebcg.html

[2] World Health Organization (WHO); United Nations Children’s Fund (UNICEF) Nigeria: WHO and UNICEF estimates of immunization coverage: 2014 revision. Geneva: WHO; 2015 Jul Available from: http://www.who.int/immunization/monitoring_surveillance/data/nga.pdf

[3] National Population Commission (NPC) [Nigeria]; ICF International Nigeria demographic and health survey 2013. Abuja (Nigeria): NPC; 201 Co-published by ICF International. Available from: http://dhsprogram.com/publications/publication-fr293-dhs-final-reports.cfm

[4] Stokes-Prindle C Wonodi C Aina M Oni G Olukowi T Pate MA Landscape analysis of routine immunization in Nigeria: identifying barriers and prioritizing interventions. Baltimore (MD): Johns Hopkins International Vaccine Access Center; 2012 Available from: http://www.jhsph.edu/research/centers-and-institutes/ivac/projects/nigeria/IVAC-Landscape-Analysis-Routine-Immunization-Nigeria-WhitePaper.pdf

[5] The World Bank Chapter 2: Sub-Saharan Africa. : Global economic prospects. Washington (DC): The World Bank; 2015 Jan p. 101–117. Available from: http://www.worldbank.org/content/dam/Worldbank/GEP/GEP2015a/pdfs/GEP2015a_chapter2_regionaloutlook_SSA.pdf

[6] Booth D Chambers V The SAVI programme in Nigeria: towards politically smart, locally led development. London: Overseas Development Institute; 2014 Available from: http://www.odi.org/publications/8876-politically-smart-nigeria

[7] Andrews M Pritchett L Woolcock M Escaping capability traps through problem driven iterative adaptation (PDIA). World Dev. 2013;51:234–244. 10.1016/j.worlddev.2013.05.011

[8] Gavi: The Vaccine Alliance [Internet] Geneva: Gavi, The Vaccine Alliance Secretariat; c2015. Country hub: Nigeria; [cited 2014 Sep 1] Available from: http://www.gavi.org/country/nigeria/

[9] Booth D Facilitating development: an arm’s length approach to aid. London: Overseas Development Institute; 2013 Available from: http://www.odi.org/publications/7376-facilitating-development-arms-length-approach-aid

[10] West GR Clapp SP Averill EM Cates W Jr. Defining and assessing evidence for the effectiveness of technical assistance in furthering global health. Glob Public Health. 2012;7(9): 915–930. 10.1080/17441692.2012.682075. . 22606939 PMC3479625

